# A new therapy in Epstein-Barr virus-associated lymphoproliferative disease: a case report and a revision of the literature

**DOI:** 10.1186/s13052-019-0741-8

**Published:** 2019-11-04

**Authors:** Lingling Xu, Hongjun Ba, Hongrong Lin, Liangying Zhong, Suping Li, Wen Tang, Zhiyong Ke, Ziyin Ye

**Affiliations:** 1 Department of Pediatric, The First Affiliated Hospital, Sun Yat-sen University, Zhongshan 2 Road, Guangzhou, 510080 People’s Republic of China; 2 Department of Laboratory Medicine pediatrics, The First Affiliated Hospital, Sun Yat-sen University, Zhongshan 2 Road, Guangzhou, 510080 People’s Republic of China; 3 Department of pathology, The First Affiliated Hospital, Sun Yat-sen University, Zhongshan 2 Road, Guangzhou, 510080 People’s Republic of China

**Keywords:** Chronic active Epstein-Barr virus infection, Thalidomide, Propranolol, Enteritis, Encephalitis, Vascular lesions

## Abstract

**Background:**

Systemic chronic active Epstein-Barr virus infection is an extremely rare childhood disease. Since chronic active Epstein-Barr virus infection can trigger the onset of Epstein-Barr virus-associated lymphoproliferative disease. The clinical manifestations of the disease vary according to the site of involvement; therefore, management may be challenging. Currently, there are no standardized guidelines for treating Chronic active Epstein-Barr virus infection effectively.

**Case presentation:**

We report a case of chronic active Epstein-Barr virus infection in a 5-year-old Chinese boy with intestinal, vascular, and neurological involvement. At age of 2 years and 7 months old, he had hepatomegaly and been diagnosed with Epstein-Barr virus infection. After treatment, he showed some clinical improvement. At age of 3 years and 3 months old, he presented with recurrent fever and diarrhea. Then he received methylprednisolone for 1 year and his symptoms ameliorated. At the age of 5 years, his symptoms recurred and had gastrointestinal hemorrhage and developed polyuria, frequent convulsions and hyponatremia. He was transferred to our hospital for further management. He was unconscious on admission and was diagnosised Epstein-Barr virus-lymphoproliferative disorder, based on the results in situ hybridization of EBV-encoded miRNA in sigmoid colon. Three-dimensional CT angiography demonstrated an aneurysm in the right internal carotid artery. Abdominal CT showed dilatation of vessels in part of the intestinal wall. He was also diagnosised Epstein-Barr virus encephalitis based on the elevated Epstein-Barr virus antibody titers and presence of Epstein-Barr virus DNA in the Cerebrospinal Fluid.

A repeated duodenal artery embolization and symptomatic therapy could not control the hemorrhage after admission. He subsequently received treatment with ganciclovir, glucocorticoid, thalidomide, and propranolol. Hemorrhage was controlled in 5 days; his symptoms improved. The fever did not recur and the CSF pressure was also normalized. A follow-up CT at 3 months after admission showed regression of the aneurysm in the right internal carotid artery and the vascular lesion in the duodenum.

**Discussion and conclusions:**

A new treatment protocol including thalidomide and propranolol resulted in a marked improvement in his clinical symptoms, and shows promise as a novel and effective therapeutic approach for Chronic active Epstein-Barr virus infection-associated lymphoproliferative disorder.

## Introduction

Chronic active Epstein-Barr virus infection (CAEBV) is a systemic EBV-positive lymphoproliferative disorder (EBV-LPD), which is marked by persistent infectious mononucleosis-like symptoms. CAEBV may result in life-threatening complications [1–6] such as malignant lymphomashepatic failure, interstitial pneumonia, coronary artery aneurysms, central nervous system (CNS) involvement, hemophagocytic syndromes and massive hemorrhage from the gastrointestinal tract. However, it is very rare for a patient to have several synchronous symptoms owing to EBV infection. Hematopoietic stem cell transplantation (HSCT) may be the only cure for CAEBV [7].

We report the case of a boy with CAEBV who synchronously developed an intestinal lymphoproliferative lesion, life-threatening gastrointestinal bleeding, multiple vascular lesions, and encephalitis. A new treatment strategy, which involved the combination of thalidomide, propranolol, ganciclovir, and glucocorticoids, successfully cured the symptoms.

## Consent

The guardian of the patient consented to treatment and also provided written consent for publication of the data in this case report.

## Case presentation

A 5-year-old boy with no personal or family history of immunodeficiency, presented with a 29 months history of intermittent fever, recurrent diarrhea and hematochezia**.** At age of 2 years and 7 months old, he had hepatomegaly and been diagnosed with EBV infection when the levels of EBV deoxyribose nucleic acid (DNA) were 7.86 ×  10^6 copies/mL (normal range: <1.0 × 10^3 copies/ml). After treatment with plasma exchange, high-dose intravenous immunoglobulins, ganciclovir, and methylprednisolone, he showed some clinical improvement.

**Fig. 1:**
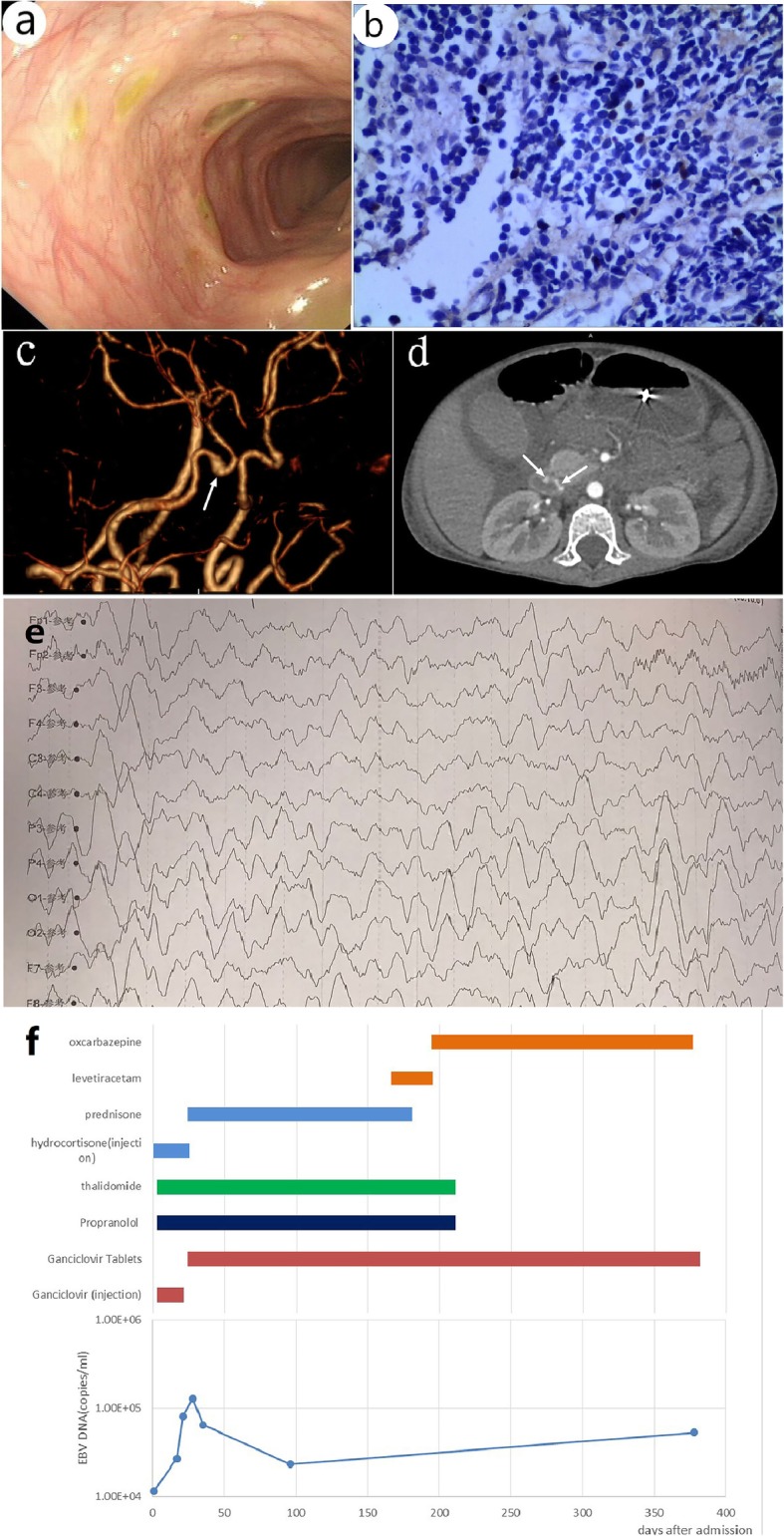
Clinical materials. **a** Colonoscopy revealed multiple and discrete ulcers as visible in the Colon; **b** EBV in situ hybridization for EBER demonstrated EBV-positive in sigmoid colon (H&E stain, 40); **c** Three-dimensional CT angiography of the head showed the cavernous segment of the right internal carotid artery presented local dilatation (white arrow); **d** Abdominal CT showed dilatation of vessels (white arrow) in part of the intestinal wall; **e** Electroencephalography showed diffuse slowing; **f** The drug therapy and Epstein-Barr virus DNA levels of the patient

At the age of 3 years and 3 months, he visited another hospital because of recurrent fever and diarrhea. Upper gastrointestinal endoscopy and enteroscopy revealed multiple ulcers in the ileum and colon (Fig. 1a). Pathological diagnosis of intestinal tract could not exclude Crohn’s disease. He was then prescribed methylprednisolone, which he continued on for 1 year with amelioration of his symptoms.

At the age of 5 years, he experienced recurrent fever, abdominal pain, and diarrhea, persisting for nearly 1 month. He also had gastrointestinal hemorrhage for 2 weeks. The laboratory results in the local hospital revealed coagulation dysfunction, anemia (the lowest hemoglobin was 62 g/L), low platelet counts (lowest count: 68 × 10^9/L), and raised procalcitonin levels (the highest level: 4.75 ng/ml). The highest serum ferroprotein level was 899 μg/L. Computed tomography (CT) of the abdomen revealed dilatation of blood vessels in the intestinal walls (Fig. 1d) and gastroscopy showed a duodenal ulcer with bleeding. He developed unexpected and sudden life-threatening hemorrhage from the intestinal vasculature, which led to hypovolemic shock. Routine management included treatment for shock, empirical antibiotics, blood transfusion, and hemostatic therapy. However, the hemorrhage was only controlled for 2 days using duodenal artery embolization. He also developed polyuria (3-4 L/d), frequent convulsions and hyponatremia (109 mEq/L). With treatment, his symptoms improved. However, the gastrointestinal hemorrhage and polyuria persisted, and the patient was transferred to our hospital for further management.

He was unconscious on admission, had a Glasgow Coma Scale (GCS) score of 11 and had hepatomegaly and splenomegaly. Laboratory tests demonstrated a white blood count of 6.02 × 10^9/L, hemoglobin level of 112 g/L, platelet count of 75 × 10^9/L, D-dimer level of 3.82 mg/L, prothrombin time of 13.3 s, activated partial thromboplastin time of 36.4 s, and a fibrinogen level of 1.66 g/L. The serum albumin levels were low at 26 g/L, but the liver function was within normal limits. The serum levels of triglyceride and ferroprotein were within normal range at 1.58 mmol/L (normal range: 0.45–1.7 mmol/L) and 227.5 μg/L, respectively (normal range: 20.0–200.0 μg/L). EBV DNA and cytomegalovirus DNA were both detected in the blood; the levels of viral copies were 1.17 × 10^4/mL and 2.62 × 10^3/mL, respectively. Tests for serum EBV antibodies, including immunoglobulin A (Ig A)/ VCA, immunoglobulin G (IgG) /VCA, and IgM/VCA, were negative. The numbers of natural killer cells in the blood were low, accounting for 0.6% of lymphocytes. Initially, the cerebrospinal fluid (CSF) pressure was abnormally raised (245 cm of H_2_O). An EBV-DNA level of 1.43 × 10^4 copies/mL was detected in the CSF by polymerase chain reaction (PCR); the CSF also tested positive for Epstein - Barr virus VCA (IgG). Three-dimensional CT angiography of the head and neck demonstrated an aneurysm in the right internal carotid artery (Fig. 1c). The echocardiography was essentially normal, without any coronary artery dilation or aneurysm. We confirmed the diagnosis of EBV-LPD, based on the results in situ hybridization of EBV-encoded miRNA (EBER) (Fig. 1b). Electroencephalo-graphy showed diffuse slowing (Fig. 1e).

## Treatment in our hospital and follow up

A repeated duodenal artery embolization and symptomatic therapy could not control the hemorrhage. He subsequently received treatment with ganciclovir, glucocorticoid, thalidomide at a dose of 2 mg/kg/d in 3 divided doses, and propranolol at an initial dose of 1.5–2 mg/kg/d. Hemorrhage was controlled in 5 days; his symptoms improved and urine output was normalized. The GCS score was 14. Then, the fever did not recur and the CSF pressure was also normalized. The level of EBV DNA of CSF was 2.5 × 10^2 copies /mL (normal value: ≤500).

He had a spontaneous remission of seizures on day 19 of admission. He was discharged from hospital on day 24 of admission. After discharge, he was diagnosed with epilepsy owing to recurring seizures, which required the successive use of levetiracetam and oxcarbazepine to control. A follow-up CT at 3 months after admission showed regression of the aneurysm in the right internal carotid artery and the vascular lesion in the duodenum. The drug therapy schedule and levels of EBV DNA are shown in Fig. 1f.

## Similar and contrasting cases in the literature

A literature search revealed only 3 reports of cases with 2 or more rare EBV-associated clinical manifestations. Mashima et al. [8] reported the case of a 55-year-old woman with aplastic anemia who was diagnosed with EBV-LPD and EBV encephalitis. Another report, by Noda et al. (2011), described the case of an immunocompetent 65-year-old man who presented with complaints of general malaise and severe disturbance of consciousness. He was initially suspected to have EBV encephalitis based on the findings on MRI of the brain, elevated EBV antibody titers, and the presence of EBV DNA in the CSF. Finally, he was diagnosed with an EBV-associated B-cell LPD with CNS involvement, and found to have EBER by in situ hybridization positivity in the brain tissue on autopsy. In the third report, Raman et al. (2014) described a patient with newly diagnosed HIV infection, who also developed cerebral vasculitis and encephalitis due to EBV.

A review of the literature is presented, with a summary of 8 cases of CAEBV-associated enteritis and EBV-LPD in non-immunocompromised individuals (Table 1).

**Table 1:** Case reports of enteritis and/or gastrointestinal haemorrhage in immunocompetent patients with EBV- LPD

Source, y	Age at diagnosis, y/Sex	Duration between symptom onset to hospital admission	Symptoms; misdiagnose	Duration of haemorrhage, amount of bleeding	Diseased region with haemorrhage	Intestinal pathology	PCR VCA-IgM VCA-IgG EA EBNA EBER	Serum EBV DNA copies/ml	Infected cell	Operation	Drug Treatment
Wang et al. [6], 2018	43y/F†	2 m	intermittent fever, chill, abdominal pain, diarrhea, hematochezia; NA	4d, large	multiple aphthous bleeding ulcers scattered from the stoma to about 40 cm away from small intestine	Multiple colonic ulcers	NT + + + + +	2.55 × 10^6	T	total colectomy	Mesalazine,Glucocorticoid,antiviral medication
Xiao et al. [9], 2016	14y/M†	1.75 y	bellyache, diarrhea, fever, hematochezia, gastrointestinal Perforations; IBD	1 y, large	Intestinal hemorrhea	diffuse heterotypic lymphoid cells infiltration, karyorrhexis and patchy necrosis	NT NT + + + +	NT	T/NK	gastrointestinal perforation repair, intestinal anastomosis	Mesalazine,Prednisone
Chen et al. [10], 2016*	29/M†*	over 1y	recurrent diarrhea,abdominal pain, fever,intestinal perforation; CD	NA,large	NA	multiple ulcers in esophagus, stomach, terminal ileum, and the entire colon	NT + NT NT NT +	NT	T	partial intestinal bowel resection,terminal ileum colostomy	Methylprednisolone, mesalazine, anti-TNF, chemotherapy
Zheng et al. [11], 2015	26y/M†	Over 3 m	intermittent fever, diarrhea, hematochezia; UC	More than 1 m, large	multiple colorectal ulcers	Multiple colorectal ulcers	+ NT + + + +	9 × 10^4	T	right hemicolectomy	antiviral and hormonal therapy
Na et al. [12], 2013	49y/F#@	19 m	recurrent hematochezia, small bowel perforation; CD	10 m, large recurrent hematochezia	small bowel	multiple ulcer scars in the cecum and ascending colon	NT NT NT NT NT NT	1.75× 10^3	T	near-total small bowel resection	Prednisolone, infliximab (5 mg/kg) infusion, chemotherapy
Na et al. [12], 2013	50y/M†	8y	weakness, anorexia, weight loss, loose stools, fever, perforation; intestinal TB and CD	No	No	an ulcer at the terminal ileum ,multiple discrete ulcers scattered from the distal ascending colon to the rectum	NT NT NT NT NT +	3.45× 10^4	T	small bowel resection; jejuno-ileostomy	anti-tuberculous, Prednisolone, Mesalamine, chemotherapy
Abdul-Ghafar et al. [13], 2011	45y/M†	45d	diarrhea, weight loss; UC	3d, large	extensive ulcerations along the whole colon	multiple ulcerations scattered along the whole colon and ileocecal valve	+ NT NT NT NT +	NT	T	total colectomy	oral metronidazole, intra-venous antibiotics
Karlitz et al. [14],2011	30y/M#	2 m	lower abdominal bloating and loose, bloody, mucoid bowel movements	NA,small	NA	diffuse erythematous and edematous mucosa located contiguously throughout the colon	+ NT NT NT NT +	NT	B	ND	supportive care alone
Our case	5y/M#	Over 2y	recurrent fever,diarrhea, abdominal pain, Hematochezia, polyuria;CD	3d,large	a duodenal ulcer and dilatation of duodenal artery	a duodenal ulcer, and the entire colon ulcers	+ - - NT NT +	1.17 × 10 ^ 4	NA	ND	Prednisolone, Ganciclovir, thalidomide, propranolol

Abbreviations: *EBV- LPD* EBV associated lymphoproliferative disorder, *PCR* polymerase chain reaction, *VCA-IgM* Viral capsid antigen Immunoglobulin M, *VCA-IgG* Viral capsid antigen Immunoglobulin G, *EA* early antigen, *EBNA* Epstein-Barr virus nuclear antigen, *EBER* EBV-encoded early small ribonucleic acid, *EBV* Epstein–Barr virus, *DNA* Deoxyribose Nucleic Acid, *F* Female, *IBD* Inflammatory Bowel Disease, *NT* not tested, *NA* not assessed, *UC* ulcerative colitis, *CD* Crohn disease, *TB* tuberculosis, *ND* not done, *+* positive test, *−* negative, *y* year or years, *m* months, *w* weeks, *d* days, *hr* hours, † Died, # Recovery, * Finally,he was diagnosed with EBV-associated NK/T-cell lymphoma.@ she was diagnosed with peripheral T-cell lymphoma

## Discussion and conclusions

The clinical manifestations of CAEBV vary according to the site of involvement, such as multiple vascular lesions, intestinal lesions, central nervous system complications and so on. A standard and effective treatment protocol for systemic EBV-LPD is lacking. HSCT is the only cure.

We report a rare case of CAEBV with intestinal, vascular, and neurological involvement. He presented a sudden life-threatening gastrointestinal hemorrhage because of enteritis and the dilatation of intestinal vasculature. It has been reported in the literature [15] that most of these conditions required surgical resection of the bowel, and if surgery was not possible, most died of massive bleeding. For our case, titanium clips and somatostatin were employed to control the hemorrhage, but it soon recurred. Interestingly, the hemorrhage was controlled within 5 days after treatment with ganciclovir, thalidomide, and propranolol. The intestinal vasculature was caused by EBV, not caused by a congenital vascular malformation, because EBER-lymphocytes were positive in the intestinal tract. A follow-up CT scan showed regression of all aneurysm. Thalidomide and propranolol are apparently effective in treating enteritis and vascular lesions secondary to EBV infection.

Both propranolol and thalidomide were known as angiogenesis inhibitor. Propranolol is the preferred treatment for accidentally diagnosed infantile hemangiomas [16, 17]. Thalidomide has proven efficacy in myeloma [18]. However, neither of these drugs have previously been used for vascular lesions associated with EBV infection.

Jones et al. [19] reported that thalidomide and pomalidomide may reactivate EBV-positive resting memory B cells, thereby enhancing the EBV lytic cycle and host immune suppression. However, thalidomide is less effective than pomalidomide in enhancing the EBV lytic cycle [19]. And patients with CAEBV may have deficiencies of EBV-specific cellular immunity, and nearly all resting memory B cells are activated. Therefore, only a few of these cells may be reactivated by thalidomide with minimal impact on the condition of these patients. Our case showed that thalidomide was safe for treating CAEBV.

Yager et al. [20] found that oral valganciclovir could inhibit EBV replication. In our patient, long-term oral ganciclovir therapy could inhibit EBV replication in the gastrointestinal tract; corticosteroids offered symptomatic relief. The improvement in the intestinal lesions in our patient confirmed this effect.

With the combination treatment, our patient’s clinical symptoms disappeared despite the persistence of EBV DNA load in peripheral blood. This proved the efficacy of this combination therapy. Our goal of treatment was not the achievement of complete remission, but long-term symptom control, regardless of the presence of the EBV genome.

We think this report and discussion may improve the understanding and management of CAEBV. This therapy may represent a safe and feasible alternative for severe CAEBV and associated LPD patients, which warrants further research.

## Data Availability

All datasets generated for this study are included in the manuscript and the supplementary files.

## Abbreviations

CAEBV: Chronic active Epstein-Barr virus infection
CD: Crohn disease
CSF: Cerebro-spinal fluid
CT: Computed tomography
DNA: Deoxyribose Nucleic Acid
EBER: EBV-encoded early small ribonucleic acid
EBV: Epstein–Barr virus
EBV-LPD: EBV associated lymphoproliferative disorder
GCS: Glasgow Coma Scale
HSCT: Hematopoietic stem cell transplantation
IgG: Immunoglobulin G
IgM: Immunoglobulin M
IM: infectious mononucleosis
VCA: Viral capsid antigen

## References

[1] Cohen JI, Jaffe ES, Dale JK, Pittaluga S, Heslop HE, Rooney CM (2011). Characterization and treatment of chronic active Epstein-Barr virus disease: a 28-year experience in the United States. BLOOD.

[2] Xing Y, Yang J, Lian G, Chen S, Chen L, Li F (2017). Chronic active Epstein-Barr virus infection associated with hemophagocytic syndrome and extra-nodal natural killer/T-cell lymphoma in an 18-year-old girl: A case report. Medicine.

[3] Rolinski J, Grywalska E, Pyzik A, Dzik M, Opoka-Winiarska V, Surdacka A (2018). Interferon alpha as antiviral therapy in chronic active Epstein-Barr virus disease with interstitial pneumonia - case report. BMC Infect Dis.

[4] Nishimura S, Ehara S, Hanatani A, Yoshiyama M (2014). Chronic active Epstein-Barr virus infection complicated with multiple artery aneurysms. Eur Heart J Cardiovasc Imaging.

[5] Kobayashi Z, Tsuchiya K, Takahashi M, Yokota O, Sasaki A, Bhunchet E (2008). An autopsy case of chronic active Epstein-Barr virus infection (CAEBV): distribution of central nervous system (CNS) lesions. J Neurol Sci.

[6] Wang Y, Li Y, Meng X, Duan X, Wang M, Chen W (2018). Epstein-Barr virus-associated T-cell Lymphoproliferative disorder presenting as chronic diarrhea and intestinal bleeding: a case report. Front Immunol.

[7] Matsui S, Takeda Y, Isshiki Y, Yamazaki A, Nakao S, Takaishi K (2016). Chronic active Epstein-Barr virus infection with marked pericardial effusion successfully treated with allogeneic peripheral blood stem cell transplantation. Rinsho Ketsueki.

[8] Mashima K, Yano S, Yokoyama H, Saito T, Machishima T, Shimada T (2017). Epstein-Barr virus-associated Lymphoproliferative disorder with encephalitis following anti-thymocyte globulin for aplastic Anemia resolved with rituximab therapy: a case report and literature review. Intern Med.

[9] Xiao HJ, Li J, Song HM, Li ZH, Dong M, Zhou XG (2016). Epstein-Barr Virus-Positive T/NK-Cell Lymphoproliferative Disorders Manifested as Gastrointestinal Perforations and Skin Lesions: A Case Report. Medicine.

[10] Chen H, Zhang Y, Jiang Z, Zhou W, Cao Q (2016). A Case Report of NK-Cell Lymphoproliferative Disease With a Wide Involvement of Digestive Tract Develop Into Epstein-Barr Virus Associated NK/T Cell Lymphoma in an Immunocompetent Patient. Medicine.

[11] Zheng X, Xie J, Zhou X (2015). Epstein-Barr virus associated T-cell lymphoproliferative disease misdiagnosed as ulcerative colitis: a case report. Int J Clin Exp Pathol.

[12] Na HK, Ye BD, Yang SK, Yang DH, Jung KW, Kim KJ (2013). EBV-associated lymphoproliferative disorders misdiagnosed as Crohn's disease. J Crohns Colitis.

[13] Abdul-Ghafar J, Kim JW, Park KH, Cho MY (2011). Fulminant Epstein-Barr virus-associated T-cell lymphoproliferative disorder in an immunocompetent middle-aged man presenting with chronic diarrhea and gastrointestinal bleeding. J Korean Med Sci.

[14] Karlitz JJ, Li ST, Holman RP, Rice MC (2011). EBV-associated colitis mimicking IBD in an immunocompetent individual. Nat Rev Gastroenterol Hepatol.

[15] Dong XY, Li J, Li Y, Wu D, Zhang Y, Cao W (2018). The clinical characteristics of immunocompetent adults with chronic active Epstein-Barr virus associated enteritis. Zhonghua Nei Ke Za Zhi.

[16] Hagen R, Ghareeb E, Jalali O, Zinn Z (2018). Infantile hemangiomas: what have we learned from propranolol?. Curr Opin Pediatr.

[17] Ji Y, Chen S, Xu C, Li L, Xiang B (2015). The use of propranolol in the treatment of infantile haemangiomas: an update on potential mechanisms of action. Br J Dermatol.

[18] Weber D, Rankin K, Gavino M, Delasalle K, Alexanian R (2003). Thalidomide alone or with dexamethasone for previously untreated multiple myeloma. J Clin Oncol.

[19] Jones RJ, Iempridee T, Wang X, Lee HC, Mertz JE, Kenney SC (2016). Lenalidomide, thalidomide, and Pomalidomide reactivate the Epstein-Barr virus lytic cycle through Phosphoinositide 3-kinase signaling and Ikaros expression. Clin Cancer Res.

[20] Yager JE, Magaret AS, Kuntz SR, Selke S, Huang ML, Corey L (2017). Valganciclovir for the suppression of Epstein-Barr virus replication. J Infect Dis.

